# LGI proteins in the nervous system

**DOI:** 10.1042/AN20120095

**Published:** 2013-06-25

**Authors:** Linde Kegel, Eerik Aunin, Dies Meijer, John R. Bermingham

**Affiliations:** *Department of Genetics and Cell Biology, Erasmus University Medical Center, PO Box 2040, 3000 CA Rotterdam, The Netherlands; †McLaughlin Research Institute, 1520 23rd Street South, Great Falls, MT 59405, U.S.A.

**Keywords:** autosomal dominant lateral temporal (lobe) epilepsy (ADLTE), autosomal dominant partial epilepsy with auditory features (ADPEAF), epitempin, leucine-rich glioma-inactivated protein, leucine-rich repeat (LRR), PNS development, synapse, ADLTE, autosomal dominant lateral temporal (lobe) epilepsy, ADPEAF, autosomal dominant partial epilepsy with auditory feature, Akt, protein kinase B, AMPA, α-amino-3-hydroxy-5-methylisoxazole-4-propionic acid, CNS, central nervous system, EPTP, epitempin, GFP, green fluorescent protein, mEPSCs, miniature excitatory postsynaptic currents, LE, limbic encephalitis, LGI, leucine-rich glioma inactivated, LRR, leucine-rich repeat, LRR-CT, carboxyl-terminal portion of the LRR, NMDA, *N*-methyl-D-aspartate, Nrg1, neuregulin, PNS, peripheral nervous system, PTZ, pentylenetetrazole, VGKC, voltage-gated potassium channel

## Abstract

The development and function of the vertebrate nervous system depend on specific interactions between different cell types. Two examples of such interactions are synaptic transmission and myelination. LGI1-4 (leucine-rich glioma inactivated proteins) play important roles in these processes. They are secreted proteins consisting of an LRR (leucine-rich repeat) domain and a so-called epilepsy-associated or EPTP (epitempin) domain. Both domains are thought to function in protein–protein interactions. The first LGI gene to be identified, *LGI1*, was found at a chromosomal translocation breakpoint in a glioma cell line. It was subsequently found mutated in ADLTE (autosomal dominant lateral temporal (lobe) epilepsy) also referred to as ADPEAF (autosomal dominant partial epilepsy with auditory features). LGI1 protein appears to act at synapses and antibodies against LGI1 may cause the autoimmune disorder limbic encephalitis. A similar function in synaptic remodelling has been suggested for LGI2, which is mutated in canine Benign Familial Juvenile Epilepsy. LGI4 is required for proliferation of glia in the peripheral nervous system and binds to a neuronal receptor, ADAM22, to foster ensheathment and myelination of axons by Schwann cells. Thus, LGI proteins play crucial roles in nervous system development and function and their study is highly important, both to understand their biological functions and for their therapeutic potential. Here, we review our current knowledge about this important family of proteins, and the progress made towards understanding their functions.

## CELL–CELL INTERACTIONS IN NERVOUS SYSTEM DEVELOPMENT

Synapse formation and maturation require multiple interactions between presynaptic and postsynaptic neurons that are mediated by a diverse set of synaptic proteins (Han and Kim, 2008; McMahon and Diaz, 2011; Siddiqui and Craig, 2011). Initial synapse formation needs both the binding of secreted proteins to presynaptic and postsynaptic receptors, and the direct binding between presynaptic and postsynaptic transmembrane proteins. Many synaptogenic proteins have been described, some of which, like the secreted LGI proteins discussed here, are specific to vertebrates.

Myelination also requires cell–cell interactions; myelin is required for rapid axonal transmission of electrical signals in the vertebrate nervous system. It consists of multiple wraps of membrane that permit saltatory conduction between specialized structures, the nodes of Ranvier. Myelination requires reciprocal interactions between the axon and oligodendrocytes in the CNS (central nervous system) or Schwann cells in the PNS (peripheral nervous system); reviewed in (Nave, 2010; Piaton et al., 2010; Quintes et al., 2010). As described below, LGI4 participates in axon-Schwann cell communication; it is crucial for PNS (but not CNS) myelination. In addition to synaptogenesis and myelination, LGI proteins are likely to participate in other cell–cell interactions as well. The study of this interesting family of proteins is critical for understanding proper development and function of the vertebrate nervous system, and for gaining insights into therapies for diseases that affect them.

## IDENTIFICATION OF THE LGI FAMILY

As suggested by its name, leucine-rich glioma inactivated, the LGI protein family was discovered in gliomas. Loss of one copy of chromosome 10 is a common event in high-grade gliomas. In a search for genes that are mutated in these gliomas, Cowell and colleagues identified a gene in the 10q24 region that was rearranged as a result of a t(10;19)(q24;q13) balanced translocation in a T98G glioblastoma multiforme cell line (Chernova et al., 1998). They suggested that the complete loss of this gene, which contains four LRRs (leucine-rich repeats), contributes to the malignant progression of glial tumours.

A completely independent line of inquiry implicated LGI1 in epilepsy. Epilepsy is a heterogeneous disease, and families with multiple epileptic individuals are important resources for identifying susceptibility genes. In one such family multiple individuals presented with partial seizures with auditory features, thereby permitting a susceptibility gene to be mapped to a 10-centimorgan region of chromosome 10 (Ottman et al., 1995). This type of epilepsy was named ADPEAF (autosomal dominant partial epilepsy with auditory features) (Winawer et al., 2000), and additional families with similar symptoms confirmed linkage to the same region of chromosome 10 (Winawer et al., 2002). Independently, a large five-generation Basque family with similar dominant partial epilepsy also demonstrated linkage to chromosome 10q (Poza et al., 1999). This inherited epilepsy was named ADLTE [autosomal dominant lateral temporal (lobe) epilepsy]. Making the assumption that the same gene was mutated in these two families reduced the chromosomal region containing the epilepsy gene to a 4.2 Mb sequence. All genes within this region were sequenced in three affected individuals of three different families revealing distinct mutations in the *LGI1* gene. Extending the analysis to two additional families revealed two more mutations (Kalachikov et al., 2002). Independently, Morante-Redolat et al. (2002) identified mutations in the *LGI1* gene in two families. Since these initial publications many more mutations in the *LGI1* gene have been found in ADLTE/ADPEAF patients and the total number is now 33 (see Ho et al., 2012). The terms ADLTE and ADPEAF refer to the same clinical entity (OMIM 600512). In this review, we use the nomenclature of Winawer et al. (2000) and refer to the disease as ADPEAF. As we discuss here, LGI1 is associated with synapses in the nervous system and most recent research has focused on the role of LGI1 in nervous system function and disease.

The other three LGI family members, *LGI2*–*3* and *-4*, were cloned following *in silico* identification of *LGI1*-homologous genes in vertebrate genomes (Gu et al., 2002). Subsequently, mutations in *Lgi2* and *Lgi4* demonstrated that these genes perform important functions in the central and peripheral nervous systems.

## EVOLUTION OF *LGI* GENES

*LGI* genes appeared in chordates [*Branchiostoma floridae* (amphioxus) has one *LGI* gene] and are found in all vertebrate genomes that have been examined to date. Early vertebrate genomes underwent two rounds of whole genome duplication (Ohno, 1970; Dehal and Boore, 2005) and presumably a single primordial *LGI* gene gave rise to four *LGI* genes. A third whole genome duplication occurred in the teleost fish lineage after the tetrapod-teleost split (Gillis et al., 2009; Van de Peer et al., 2009; Manning and Scheeff, 2010). The zebrafish, *Danio rerio*, a teleost, possesses two copies of *lgi1* (*lgi1a* and *lgi1b*), two copies of *lgi2* (*lgi2a* and *lgi2b*), a single copy of *lgi3* and no copy of *lgi4* (Gu et al., 2005b). A similar picture–with duplicated *lgi1* and *lgi2* genes, a single *lgi3* gene and no *lgi4* gene–emerges from the genomes of other teleost fishes such as cod, stickleback and platyfish (see the webpage of Ensembl.org). Following whole genome duplication events, gene families often contract, as genes with redundant functions are lost (Manning and Scheeff, 2010). In the case of fishes, *lgi4* may have been lost as one or more other *LGI* genes assumed its functions in peripheral nerve development. Alternatively, *lgi4* diverged from an ancestral *LGI* gene specifically in the tetrapod lineage. Coelacanths (*Latimeria* sp.) are an order of fish that diverged from teleost fishes prior to their third genome duplication and are thus more closely related to the ancestors of the tetrapods. The coelacanth genome (Amemiya et al., 2013) contains single copies of *lgi1*, *lgi2*, and *lgi3* genes, but not *lgi4*. Surprisingly, the recently sequenced *Xenopus tropicalis* genome (Hellsten et al., 2010) has been found to contain *lgi1*, *lgi2* and *lgi4*, but not *lgi3*. Although it remains to be seen if the absence of individual *LGI* genes in coelacanth and frog genomes is real, the current genome sequence data support the appearance of *Lgi4* in tetrapods. As more genomes are sequenced, the pattern of *LGI* gene evolution will become clearer.

## STRUCTURE OF LGI PROTEINS

**Figure 1:**
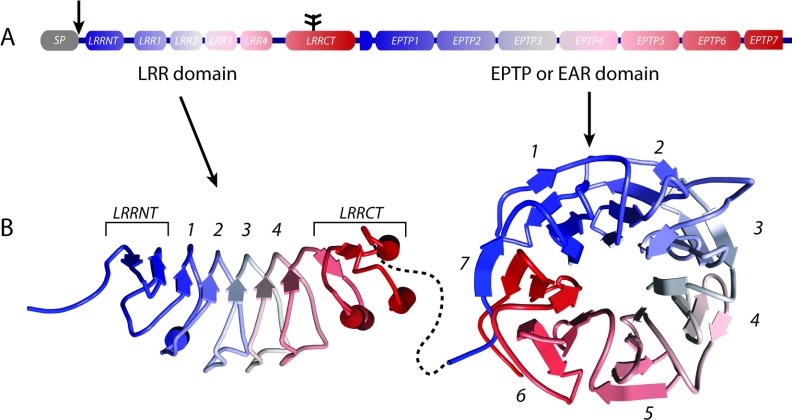
Structural characteristics of the LGI protein family In (**A**), the general domain structure of the LGI protein family is depicted. All LGI proteins have an SP (signal peptide) that is cleaved off (arrow in **A**) and is not included in the putative protein structures shown in (**B**). The glycosylation site present in all LGI members is indicated in (**A**) with a branched line structure. The putative structures of the LRR domain and EPTP domain were predicted separately using the HHpred tool (http://toolkit.tuebingen.mpg.de/hhpred) and the WDR5 protein structure (PDB 2GNQA) as template. Structures were visualized using the Accelrys Discovery Studio visualizer. The structure is colour-coded from N-terminus (blue) to C-terminus (red) and corresponds with the colour code in (**A**). The colour-code graphically reveals the Velcro β-strand (blue) interacting with the last β-strand (red) of the seventh EPTP module to zip up the EPTP domain structure. How the LRR domain and the EPTP domain are oriented towards each other is unknown. The stippled black line does not represent any structural feature but is only intended to show the linkage between the two domains.

All LGI family members have a calculated molecular mass of approximately 60 kDa (Chernova et al., 1998) and contain a signal peptide that is cleaved off, an LRR domain containing four LRR repeats flanked by cysteine-rich sequences (Kobe and Kajava, 2001) and an EPTP domain consisting of seven EPTP repeats (Figure 1A) (Scheel et al., 2002; Staub et al., 2002). The EPTP repeats most likely fold into a so-called seven bladed β-propeller, a structure that resembles a slightly conical doughnut. Both the LRR domain and the β-propeller structure provide a scaffold for specific protein interactions and are found in a wide range of proteins with diverse physiological function (Buchanan and Gay, 1996; Paoli, 2001b), but only a handful of LGI protein binding partners have been identified (see below). Some of these proteins interact with all LGI proteins, whereas others bind more selectively (Özkaynak et al., 2010; Thomas et al., 2010) and (E. Aunin, unpublished work). These proteins will be discussed in the context of the individual LGI proteins to which they bind.

The LGI proteins’ LRR domain is most homologous to Slit proteins (Krex et al., 2002). Slit proteins are large secreted proteins involved in axonal guidance and neuronal migration through interaction with their receptor Roundabout (Robo). The Slit proteins contain four LRR domains, D1–D4, and each domain is approximately 31–33% homologous to the LGI LRR domain. A model of the LGI1 LRR domain (Leonardi et al., 2011), based on the structure of Slit LRRs, suggests that it consists of four 24 amino acid long LRRs. The intron–exon structure of *LGI* genes further corroborates this conjecture as the repeats are encoded by four 72 nt long exons (exon 2 to 5). The LRRs with the N- and C-terminal flanking sequences form a slightly curved structure with parallel β-strands forming the concave face of the domain (Figure 1B). As Slit proteins dimerize (Howitt et al., 2004), one hypothesized function of the LRR domain is dimerization. Indeed, LGI1 is secreted as an oligomer (Fukata et al., 2006) raising the possibility that the LGI proteins homo- or heterodimerize with other LGI proteins or possibly with other LRR-containing proteins.

The EPTP domain comprises the C-terminal three/fifths of LGI proteins. The name Epitempin derives from the observation that this domain is found in two epilepsy-associated proteins, LGI1 and GPR98/VLGR/MASS, the latter a transmembrane protein mutated in human and mouse auditory epilepsy (Skradski et al., 2001; Nakayama et al., 2002; McMillan and White, 2004). This name is a bit misleading as LGI3 and -4, and the deafness-associated protein TSPEAR (Delmaghani et al., 2012), also contain EPTP repeats but have not yet been associated with epilepsy. Notably, mutations in the EPTP-encoding genes *LGI1*, *GPR98* and *TSPEAR* all produce auditory symptoms, either noise-induced seizures or deafness. As mentioned, the EPTP repeats of LGI proteins likely form a seven bladed β-propeller domain (Scheel et al., 2002; Staub et al., 2002). The β-propeller domain consists of four to ten modules (the blades), and is found in a wide variety of proteins in both prokaryotes and eukaryotes (Fulop and Jones, 1999; Jawad and Paoli, 2002; Pons et al., 2003; Chaudhuri et al., 2008). Each β-propeller module consists of four antiparallel β-strands that form a sheet warped like a propeller blade (see Figure 1B). In the case of the EPTP domain, seven of these modules associate to form a disc with a conical central pore, and stabilized by hydrophobic interactions between the blades (see Figure 1B). Sites for interactions with other proteins reside on the outer surfaces, and for some β-propellers, the pore contains a substrate-binding site, but whether or not anything associates with the central pore of LGI proteins is unknown.

Recently, *in silico* model(s) of the LGI1 EPTP domain have been published, based on the structure of the β-propeller protein WDR5 (Limviphuvadh et al., 2010; Leonardi et al., 2011). A notable feature of the WDR5 structure is that the seven clusters of four β-strands, seen in the primary sequence, are out of register with the seven blades in the structure (reviewed in Fulop and Jones, 1999; Xu and Min, 2011). Accordingly, the register of β-strands in the *in silico* model of LGI1 structure proposed by Leonardi et al. (2011) is shifted by one β-strand relative to the boundaries of each EPTP repeat that were described initially (Scheel et al., 2002; Staub et al., 2002). A characteristic of β-propeller domains with six or more repeats is the presence of a ‘Velcro’ or ‘molecular clasp’, sequence that holds the circular structure closed (reviewed in Paoli, 2001a). These Velcro sequences consist of one or more β-strands contributed by one terminal repeat module that are integrated into the β-sheet formed by the repeat at the other end, permitting inter-β-strand interactions to pull the entire β-propeller closed, thereby stabilizing its circular structure. Leonardi and colleagues propose that for LGI1, the N-terminal EPTP β-strand resides at the outside of the C-terminal β-propeller, with the C-terminal β-strand nested inside it (Figure 1B). The Leonardi model provides a plausible explanation for the deleterious phenotype of the 1639insA mutation in one family of ADPEAF patients (Kalachikov et al., 2002), and the LGI2 truncation seen in BJFE dogs (Seppälä et al., 2011). These mutations replace or remove the 11 C-terminal amino acids of LGI1 or LGI2, respectively. Their absence may preclude formation of the first EPTP domain, and preclude folding of the entire β-propeller structure.

How the LRR and EPTP domains of LGI proteins are oriented relative to one another is not known. Currently, this important question cannot be answered by modelling alone. The crystal structure of an intact LGI protein is needed to provide insight into the specific functions of the four LGI proteins, and to provide better understanding of the effects of the different human *LGI1* mutations.

## POST-TRANSLATIONAL MODIFICATION AND SECRETION OF LGI PROTEINS

All LGI proteins possess consensus *N*-linked glycosylation sites (Figure 1A). Asn^192^, located within the LRR-CT (C-terminal portion of the LRR) domain, resides in a glycosylation site that is conserved among all LGI members (Figure 1). Asn^277^ is part of a glycosylation site in some LGI1 and LGI2 orthologues, and a glycosylation site that includes Asn^422^ is found only in mammalian LGI1 proteins. These sites have been demonstrated to be glycosylated in LGI1 (Sirerol-Piquer et al., 2006). A triple glycosylation mutant of LGI1 is not secreted and secretion of the N192Q mutant is severely diminished, underscoring the importance of these glycosylation sites for normal maturation and secretion of LGI1. LGI4 is glycosylated at the LRR-CT site (Bermingham et al., 2006), but the functional significance of this is unknown. Based on the observations with LGI1, it is reasonable to postulate that LGI proteins require glycosylation for one or more steps in their secretion.

Most ADPEAF mutations inhibit LGI1 secretion (de Bellescize et al., 2009; Di Bonaventura et al., 2011; Striano et al., 2011); see Table 1 in (Nobile et al., 2009). Many of these appear to alter the ability of the LRR domain or the EPTP domain to fold properly, thereby inhibiting their secretion. Accordingly, these mutations have provided limited information about the functional interactions of LGI1 protein. However, one ADPEAF mutation, Arg^407^Cys is secreted normally (Striano et al., 2011). Rather than affecting LGI1 protein stability or secretion, this mutation may alter a functionally significant interaction domain for LGI1, and provides a proof of principle for utilizing LGI1 structural information to design mutations that will inform us about LGI1 function.

**Table 1:** Potential binding partners of Lgi proteins Proteins whose association with LGI proteins have been tested directly by co-immunoprecipitation or by co-localization of tagged proteins in cultured cells, are shown. References are abbreviated as follows: F06 (Fukata et al., 2006); F10 (Fukata et al., 2010); K (Kim et al., 2012); N (Nishino et al., 2010); Ok (Okabayashi and Kimura, 2010); Oz (Özkaynak et al., 2010); P (Park et al., 2008); Sa08 (Sagane et al., 2008); Sa10 (Sagane et al., 2010); Sp (Seppälä et al., 2011); T (Thomas et al., 2010); W (Owuor et al., 2009). In addition, LGI1 has been shown ***not*** to bind to ADAM12 (W), ROBO2 (F06), Stargazin (F06), NGR2(T), NGR3(T), and Kv1.1(F10). ADAM9 does not bind to LGI1 (F06, F10) or to LGI4 (N).

Binding partner	Lgi1	Lgi2	Lgi3	Lgi4
ADAM11	Weak (Sa08)	Binds (Oz)	Does not bind (Oz)	Moderate (Sa08)
	Binds (W, F10)			Binds (Oz, N)
ADAM22	Moderate (Sa08)	Does not bind (Oz)	Binds (Oz)	Strong (Sa08, Sa10)
	Binds (T, W, F06; F10)	Binds (Sp)	Does not bind (F10)	Binds (Oz, N)
ADAM23	Strong (Sa08)	Does not bind (Oz)	Binds (O)	Strong (Sa08)
	Binds (T, W, F06, F10)	Binds (Sp)	Binds (K)	Binds (Oz, N)
FLOTILLIN1	ND	ND	Binds (Ok)	ND
SYNTAXIN1	ND	ND	Binds (P)	ND
NGR1	Binds (T)	ND	ND	ND

## LGI1 MUTATIONS IN ADPEAF

**Figure 2:**
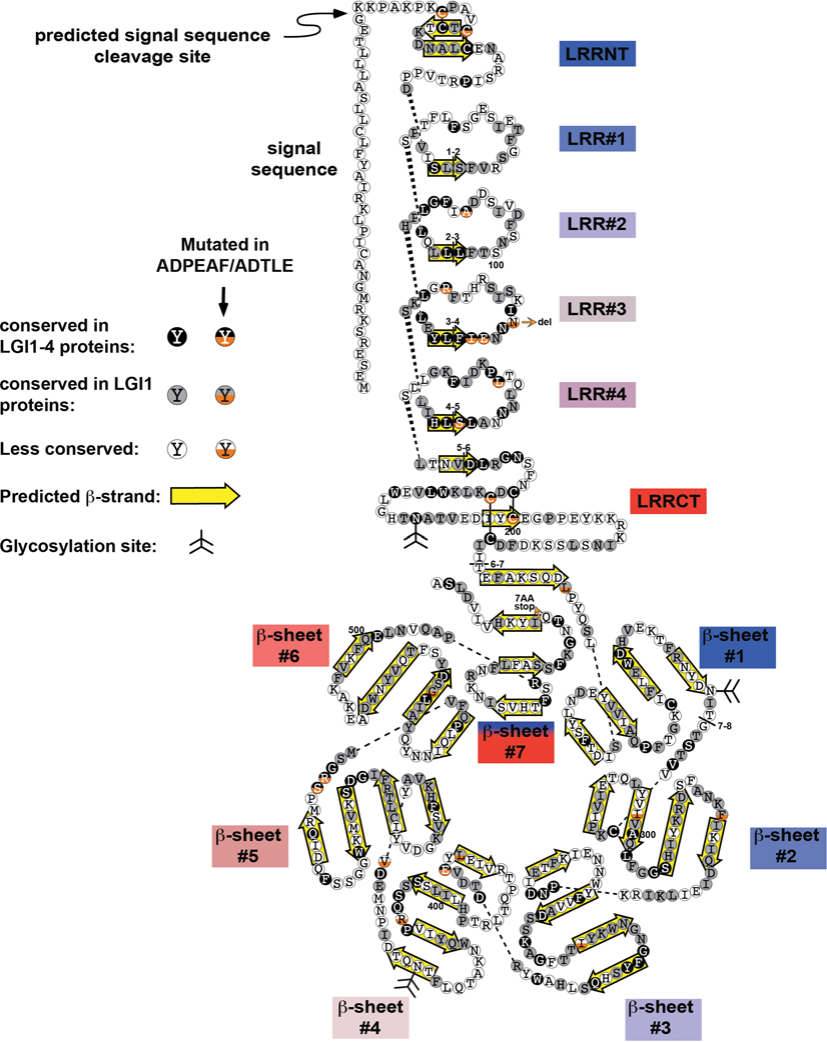
A schematic diagram of human LGI1 protein In this ‘exploded’ view of LGI1, the individual LRR and EPTP modules are separated from one another; dashed lines connect amino acids that are linked in the intact protein. Amino acids are represented as filled, shaded or open circles, depending on their level of conservation. The bottom half of circles for amino acids that are changed by point mutations are coloured orange; all of these mutations are from human except for the mutation at Leu^385^, which is from rat. For simplicity, frameshift mutations are omitted, with the exception of one at the C-terminus that provides evidence that the ‘Velcro’ model of β-propeller closure, in which the N-terminal β-strand is included in the seventh EPTP propeller fold. Human LGI1 mutations were obtained from (de Bellescize et al., 2009; Nobile et al., 2009; Di Bonaventura et al., 2011; Ho et al., 2012) and the rat mutation from (Baulac et al., 2012). For the EPTP domain, the β-propeller blades, which in large part would intersect the plane of the figure in the intact protein, have been laid flat. β-strands are outlined by yellow arrows (Leonardi et al., 2011). Disulfide bonds are depicted as solid lines, and glycosylation sites have a branched line structure. Boundaries of the eight *LGI1* exons are presented as lines through the sequence, with the relevant exon numbers juxtaposed. Every 100th amino acid is labelled.

As mentioned earlier, LGI1 mutations result in ADPEAF (OMIM 600512) (Kalachikov et al., 2002). ADPEAF patients present with complex partial and secondarily generalized seizures that are often associated with auditory auras (Nobile et al., 2009; Michelucci et al., 2013). The average age of seizure onset is in early adulthood (Winawer et al., 2000) and subtle abnormalities have been observed by magnetic resonance imaging from ADPEAF patients (Kobayashi et al., 2003; Tessa et al., 2007). This suggests that ADPEAF results from defects in brain development, which is consistent with the hypothesized functions for LGI1. ADPEAF mutations have begun to be examined in the context of other neurological diseases. LGI1 mutations do not appear to affect depression independently of epilepsy (Heiman et al., 2010). The LGI1 mutation in one ADPEAF family has been correlated with hyperactivity (Berghuis et al., 2013), and it will be important to determine if LGI1 mutations impact other diseases of synaptic connectivity. To date, 33 mutations at 29 distinct locations have been identified in LGI1 with an average penetrance of approximately 67% (Rosanoff and Ottman, 2008; Nobile et al., 2009; Kawamata et al., 2010; Michelucci et al., 2013) (Figure 2). Not all ADPEAF patients possess mutations in LGI1; the penetrance of ADPEAF in families that segregate LGI1 mutations is higher than in ADPEAF families without LGI1 mutations (Michelucci et al., 2013), suggesting that the disease has a complex and heterogeneous pattern of inheritance.

The dominant LGI1 mutations in ADPEAF could be either haploinsufficient (one wild-type copy makes insufficient amounts of protein for proper function) or dominant negative (i.e. mutant LGI1 interferes with wild-type LGI1 or its binding partners). The incomplete penetrance of many ADPEAF mutations, and the instability and/or inability to be secreted of many LGI1 proteins resulting from them suggests that these mutations are haploinsufficient (Senechal et al., 2005; Sirerol-Piquer, 2006). However, the observation that LGI proteins multimerize (Fukata et al., 2006) suggests a potential for some LGI1 mutations to exert dominant negative effects. Futhermore, overexpression of a truncated LGI1 (835delC, which corresponds to a truncation of the C-terminus of the LRR-CT and the entire EPTP domain) inhibited dendritic pruning *in vivo*, suggesting that this ADPEAF mutation has a dominant negative effect (Zhou et al., 2009). Some of the potential dominant negative effects may be attributable to endoplasmic reticulum stress and activation of the unfolded protein response, similar to what has been observed elsewhere (D’Antonio et al., 2013; Li et al., 2013; Roussel et al., 2013). Curiously, LGI1 was identified in a screen for genes with monoallelic expression (Wang et al., 2010), suggesting that such expression may explain the incomplete penetrance of some ADPEAF mutations, or the haploinsufficiency of others. Multiple mechanisms may produce the dominant phenotype of LGI1 mutations.

## LGI1 PROTEIN INTERACTIONS IN THE CNS

**Figure 3:**
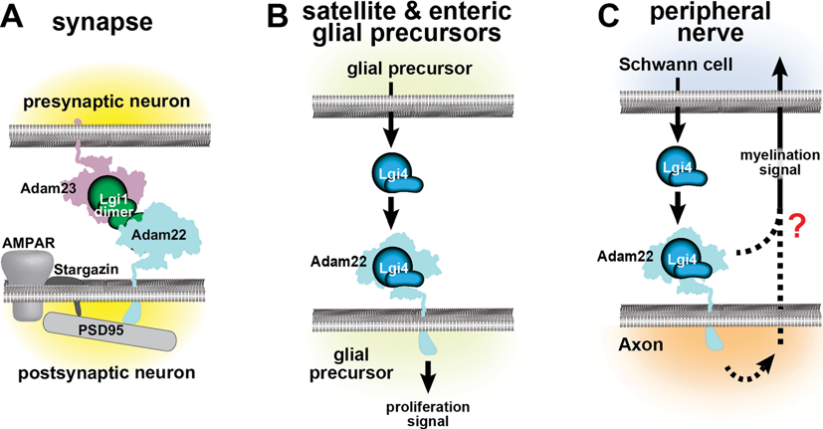
Possible mechanisms by which LGI proteins participate in cell–cell interactions Panels (**A–C**) depict several possible mechanisms by which LGI1 controls synapse development and function, and LGI4 controls PNS development. Note that these mechanisms are not mutually exclusive; the figure simply summarizes what currently is known or postulated for each interaction. (**A**) At synapses, LGI1 dimers may form a bridge between post-synaptic ADAM22, which is linked to the stargazin/AMPAR complex through PSD95 and stargazin, and presynaptic ADAM23 (Fukata et al., 2010). LGI1 has an effect on Kv1.1 inactivation through mechanisms that are unclear (Fukata et al., 2006). (**B**) In peripheral glial precursors, LGI4 appears to act as a paracrine or autocrine factor through binding to ADAM22 (Nishino et al., 2010). (**C**) During Schwann cell ensheathment and myelination of axons, LGI4 binds to the disintegrin domain of ADAM22. This interaction could trigger signalling or protein localization within the axon, thereby activating signal(s) to the Schwann cell. An LGI4–ADAM22–PSD95 interaction could cluster proteins at the axonal membrane. Alternatively, LGI4 binding to ADAM22 could induce the formation of protein complex(es) extracellularly. LGI4 might modulate ADAM22–integrin interactions, which also utilize the ADAM22 disintegrin domain (D’Abaco et al., 2006).

All LGIs seem to interact with select members of the ADAM (A Disintegrin And Metalloprotease) transmembrane protein family (Table 1). More than 40 ADAMs have been identified in species from *Caenorhabditis elegans* to human. Some members are catalytically active metalloproteases and control cell signalling by activating membrane-bound growth factors or by shedding the ectodomain of cell-surface receptors (Seals and Courtneidge, 2003; Blobel, 2005). Other members are inactive and are thought to be involved in protein interactions, especially with integrins (D’Abaco et al., 2006). LGI1 binds to the extracellular domain of ADAM22, which binds to the third PDZ domain of PSD-95 through its cytoplasmic C-terminal ETSI-motif (Fukata et al., 2006). The first two PDZ domains of PSD-95 in turn bind to the C-terminal tail of stargazin, a transmembrane regulatory subunit of AMPA (α-amino-3-hydroxy-5-methylisoxazole-4-propionic acid)-receptors that is critical for AMPA-receptor trafficking and gating (Chen et al., 2000; Tomita et al., 2005; Nicoll et al., 2006; Yokoi et al., 2012). In addition to the stargazin/AMPA receptor complex, Fukata's group found that PSD-95 strongly associated with LGI1 and ADAM22 in rat brain (Fukata et al., 2006). Thus, PSD-95 may tether two protein complexes, stargazin/AMPAR and ADAM22/LGI1 (Figure 3A). Since PSD95 together with stargazin controls the number of AMPA receptors at synapses, this association could explain the increase of synaptic AMPA/NMDA (*N*-methyl-D-aspartate) ratio in hippocampal slices after incubation with LGI1; it suggests that LGI1 is an extracellular factor-controlling synaptic strength at excitatory synapses (Fukata et al., 2006). Additionally, interaction between ADAM23 and LGI1 has been identified in the brain (Fukata et al., 2010) and on cultured neurons (Owuor et al., 2009). Both ADAM22 knockout and ADAM23 knockout mice show strong overlap in phenotype with LGI1 knockout mice, which is characterized by severe spontaneous epilepsy and premature death (Sagane et al., 2005; Owuor et al., 2009; Chabrol et al., 2010; Fukata et al., 2010; Yu et al., 2010). Additionally, a point mutation in Adam23 has been found in epileptic Belgian Shepherd dogs (Seppälä et al., 2012). Importantly, ADAM22 and ADAM23 co-assemble in the brain dependent on LGI1 (Fukata et al., 2010). Thus, it was hypothesized that LGI1 forms a bridge between presynaptic ADAM23 and postsynaptic ADAM22 (Fukata et al., 2010) (Figure 3) effectively regulating trans-synaptic interactions contributing to synaptic strength.

In addition to ADAM22 and ADAM23, LGI1 also binds to ADAM11 (Sagane et al., 2008), which is an ADAM protein essential for spatial learning, motor coordination and nociceptive responses (Takahashi et al., 2006a, 2006b). However, the question whether ADAM11 resides within the same complex with LGI1, ADAM22 and ADAM23 was not addressed and electro-physiological studies will be needed to determine if ADAM11 has a role in synaptic transmission and plasticity. Furthermore, the interaction between ADAM23 and LGI1 affects neurite outgrowth. This conclusion is based on the observation that addition of LGI1 to DRGs or hippocampal neurons causes a dose dependent increase in neurite outgrowth, and this effect is reduced for neurons cultured from *Adam23* knockout mice (Owuor et al., 2009).

LGI1 protein appears to affect NMDA-receptor subunit expression. The NMDA receptor (NMDA-R) forms a hetero-tetramer between two NR1 and two NR2 subunits. A hallmark of glutamatergic synapse maturation is the change in postsynaptic NMDA-R NR2 subunit composition (Barth and Malenka, 2001; Waites et al., 2005). The NR2B subunit is mainly expressed in immature neurons of the early postnatal brain. During development, the number of NR2A subunits grows, and eventually NR2A subunits outnumber NR2B subunits. LGI1 expression *in vivo* increases when the NR2B/NR2A ratio decreases, and when mutant ADPEAF LGI1 is overexpressed in mice, NR2B/NR2A increases (Zhou et al., 2009). These observations suggest that LGI1 regulates postsynaptic function during development. The same study showed that overexpressed ADPEAF mutant LGI1 blocks the normal developmental pruning of excess neuronal dendrites, resulting in an increase of excitatory synaptic transmission and seizure susceptibility. Both LGI1 and integrins (Chavis and Westbrook, 2001) seem to be crucial to the synchronous maturation of pre- and postsynaptic membrane functions of glutamatergic synapses during postnatal development and both bind the same disintegrin domain of ADAM proteins (Fukata et al., 2006; Yang et al., 2006). Furthermore, both integrin and LGI1 regulate NR2 subunit composition via tyrosine kinase signalling (Chavis and Westbrook, 2001; Zhou et al., 2009). These observations suggest that LGI1, integrins and ADAM proteins may cooperate to promote glutamatergic synapse maturation.

LGI1 also indirectly binds Kv1.1 VGKC (voltage-gated potassium channels), which is a major constituent of presynaptic A-type channels that modulate synaptic transmission in CNS neurons (Schulte et al., 2006). LGI1 protein expression was reported to reduce Kv1.1 inactivation via the intracellular β-subunit of the channel (Schulte et al., 2006). However, since LGI1 has a signalling sequence and is secreted, it is unlikely that LGI1 competes with Kvβ1 for binding to Kv1.1. In contrast to wild-type LGI1 protein, certain mutant LGI proteins that cause ADPEAF in patients could not reduce Kv1.1 inactivation (Schulte et al., 2006). Interestingly, these same mutant LGI proteins are not secreted (Senechal et al., 2005; Sirerol-Piquer et al., 2006; Nobile et al., 2009) suggesting that LGI1 needs to be secreted prior to having an effect on channel inactivation.

Since the LRR domains of LGI proteins are very homologous to the Slit proteins, they might have similar functions. Indeed, LGI1 counteracts myelin-induced growth cone collapse and neurite outgrowth inhibition (Thomas et al., 2010). LGI1 binds to Nogo receptor 1 (NgR1), raising the possibility that LGI1 is an antagonist of myelin-based growth inhibitors (Thomas et al., 2010). ADAM22 resides in a complex with NgR1 and facilitates LGI1 binding to this receptor, suggesting that NgR1 and ADAM22 collaborate to create an LGI1 binding complex that is important for synapse formation (Thomas et al., 2010). It has not been tested whether other LGI proteins bind any of the Nogo receptors. Together these observations underscore an important role for LGI1 in brain excitation and development, explaining why LGI1 mutations result in epilepsy.

## THE ROLE OF LGI1 IN CNS DEVELOPMENT AND FUNCTION

In the CNS, LGI1 expression patterns are complex. Although the LGI1 gene is active in the caudal ganglionic eminence at e13.5 in the mouse (Kusuzawa et al., 2012), it appears to be expressed primarily postnatally. It is expressed in multiple, discrete locations in adult brain (Kalachikov et al., 2002; Morante-Redolat et al., 2002; Senechal et al., 2005; Magdaleno et al., 2006; Ribeiro et al., 2008; Yu et al., 2010). Studies using transgenic mice that possess a bacterial artificial chromosome in which *Lgi1* regulatory sequences drive expression of GFP (green fluorescent protein) have produced similar results (Head et al., 2007). In these mice, *Lgi1*-driven GFP expression is observed in glial cells in some brain regions, whereas in others, GFP expression was exclusively neuronal. The observation of *Lgi1*-driven GFP expression in glial cells of the midbrain and elsewhere is significant in light of the role of the midbrain in the propagation of audiogenic seizures (Garcia-Cairasco et al., 1993; Garcia-Cairasco, 2002; Doretto et al., 2009). The importance for normal CNS function of LGI1 expression in specific cell types and brain regions remains to be determined.

Mice and rats that lack *Lgi1* die of seizures during the third postnatal week, showing that normal development or function of the CNS requires LGI1 (Chabrol et al., 2010; Fukata et al., 2010; Yu et al., 2010; Baulac et al., 2012). Two of the three independently created lines of *Lgi1* knockout mice have been subjected to electrophysiological analyses. mEPSCs (miniature excitatory postsynaptic currents) result from the spontaneous release of synaptic vesicles in the absence of a stimulus; changes in their frequency suggest a presynaptic defect, whereas changes in their amplitude suggest a postsynaptic defect. In brain slices from their *Lgi1* mutant mice, Fukata et al. (2010) found a decrease in mEPSC amplitude but no change in frequency, suggesting that LGI1 functions postsynaptically. In contrast, Yu et al. (2010) observed an increase in the frequency of mEPSCs with no differences in their amplitudes using brain slices from their *Lgi1* knockout mice. Thus, despite the similar seizure phenotypes of their mouse strains, the groups came to opposite conclusions as to whether LGI1 acts pre- or postsynaptically. Other electrophysiological studies on BAC transgenic mice overexpressing either wild-type *Lgi1* or a mutant form that is found in ADPEAF, suggest that LGI1 acts both pre- and postsynaptically (Zhou et al., 2009). The apparent paradox may result from subtle differences in complicated experimental procedures, or more interestingly, it might reflect different activities that occur *in vivo*. Mice expressing mutant LGI1 also display reduced developmental pruning of dendritic arbors of hippocampal granule cells and increased spine density, thereby increasing neural excitability (Zhou et al., 2009). Similarly, it was demonstrated recently that LGI1 also regulates the pruning of retinogeniculate fibres (Zhou et al., 2012). These observations are consistent with increased neuronal outgrowth of wild-type mouse neurons by Lgi1 (Owuor et al., 2009). Thus, LGI–ADAM complexes add to a growing list of trans-synaptic complexes whose precise role in synaptic maturation and preservation are yet to be elucidated (reviewed in McMahon and Diaz, 2011; Siddiqui and Craig, 2011).

## *Lgi1* MUTATIONS IN ZEBRAFISH AND RATS

Rats and zebrafish with mutations in their LGI1 homologues are important resources for the development of new treatments for ADPEAF and perhaps other epilepsies as well. Expression of both homologues of *LGI1* in zebrafish, *lgi1a* and *lgi1b*, has been knocked down using antisense morpholinos, demonstrating that the two genes have acquired distinct but overlapping developmental functions (Teng et al., 2010, 2011). Low-level inhibition of *lgi1a* has no effect on fish morphology, but sensitizes them to the seizure-inducing drug PTZ (pentylenetetrazole). High-level inhibition of *lgi1a* produces developmental abnormalities, including reductions in brain, eyes and tail, presumably due to increased CNS apoptosis; these fish also display seizure-like hyperactive swimming behaviours (Teng et al., 2010). In contrast, *lgi1b* knockdown fish display hydrocephalus and heart oedema, but not hyperactivity, yet retain sensitivity to PTZ (Teng et al., 2011). Rats that carry an ENU (*N*-ethyl-*N*-nitrosourea)-generated leucine to arginine mutation (L385R) in the fourth EPTP domain of LGI1 recapitulate the susceptibility to audiogenic seizures seen in ADPEAF, and display a similar profile of anti-epileptic drugs that suppress those seizures (Baulac et al., 2012). The Zebrafish *lgi1* mutants will permit screens for small molecules that ameliorate their abnormal phenotypes, whereas the *Lgi1* mutant rats will be useful for preclinical testing of candidate antiepileptic drugs.

## LGI1 IN GLIOMAS AND OTHER CANCERS

Although LGI1 was identified initially from chromosomal breakpoint(s) in high-grade gliomas, and was proposed to function as a tumour suppressor gene (Chernova et al., 1998), its role in oncogenesis remains controversial. LGI1 expression is reduced or absent in many glioma cell lines (Chernova et al., 1998; Krex et al., 2002; Rossi et al., 2005); it also has been reported to be down-regulated in glioma tumours (Besleaga et al., 2003), in Barrett’s-related adenocarcinoma of the esophagus (Peng et al., 2008), and in prostate cancer (Cowell et al., 2010). LGI1 expression in neuroblastoma cells inhibits proliferation and causes apoptosis (Gabellini et al., 2006), further suggesting its anti-oncogenic potential. In glioma cells, LGI1 re-expression reduces their ability to proliferate and form colonies on soft agar in one study (Kunapuli et al., 2003), but not in another (Krex et al., 2002). In contrast, co-expression of a neuronal marker and LGI1 in gliomas suggests that LGI1 expression levels in these tumours may relate to the number of trapped neurons (Piepoli et al., 2006), and ADPEAF patients do not show increased frequencies of glioma (Brodtkorb et al., 2003; Gu et al., 2005a). An important use of *Lgi1* knockout mice will be to assess their susceptibility to tumorigenesis. Additionally, the effect of LGI1 expression on increasing AKT (protein kinase B) signalling, reducing ERK (extracellular-signal-regulated kinase) signalling, and reducing matrix metalloproteinase expression in glioma and other cells in culture (Kunapuli et al., 2004; Sirerol-Piquer et al., 2006; Kunapuli et al., 2010) may provide clues about its function *in vivo*.

## LGI1 AND LIMBIC ENCEPHALITIS

LE (limbic encephalitis) is a neurological autoimmune disease associated with antibodies against a variety of antigens (reviewed in Tuzun and Dalmau, 2007; Irani and Vincent, 2011; Vincent et al., 2011). Symptoms include memory loss, confusion, brain MRI abnormalities and seizures. Recently, in such patients auto-immune antibodies against LGI1 have been found (Irani et al., 2010; Lai et al., 2010). As the seizure susceptibility of LE patients is also observed in ADPEAF patients and *Lgi1* mutant rats and mice, it is reasonable to assume that at least some of the LE symptoms result from a reduction in LGI1 levels. Initially, LE was considered rare and tumour-associated. Currently, it is recognized also in patients free of tumours, to present with a variety of symptoms, and to involve tissues beyond the limbic system. VGKCs are a common autoimmune antigen in these patients. Anti-VGKC antibodies typically are detected by their ability to immunoprecipitate radio-labelled α-dendrotoxin, a potassium channel-binding protein, after it is added to lysates of brain tissue. However, upon further study, it was found that most of these antibodies reacted with LGI1 and not with potassium channel subunits (Irani et al., 2010; Lai et al., 2010). In fact, almost 90% of these LE cases possess LGI1-reactive sera. Some cases of LE are preceded by (a prodrome) or overlap with faciobrachial dystonic seizures, which are characterized by frequent brief seizures that typically affect an arm and ipsilateral face (Irani et al., 2011). Recognition of these clinical signs as a prelude to full LE provides a time window for early therapeutic intervention to limit the severity of LE symptoms and maybe prevent permanent disability. These clinical observations confirm that LGI1 is essential for proper functioning of vertebrate synapses, not only for their maturation. Epilepsy affects 1% of people worldwide; many of these cases are idiopathic and may have an autoimmune aetiology. Therefore the low prevalence of ADPEAF may understate the importance of *LGI1* as an epilepsy gene. Should pathogenic anti-LGI1 antibodies bind to specific epitopes, these epitopes could be druggable and lead to effective new epilepsy treatments.

What can anti-LGI1-mediated LE tell us about LGI1 function? First, the onset of pathology in LE patients suggests that LGI1 is required for functioning of fully developed synapses, in addition to its role in synaptic maturation (Owuor et al., 2009). The differences between the symptoms of ADPEAF and LE patients may reflect disruption in both developing and mature synapses in the former, versus only mature synapses in the latter. Secondly, faciobrachial dystonia likely involves the basal ganglia (Irani et al., 2010; Plantone et al., 2013), suggesting that *Lgi1* expression there (Head et al., 2007) is functionally significant. Thirdly, patient anti-LGI1 antibodies trigger epileptiform activity in hippocampal slices (Lalic et al., 2011). This experimental model system will help identify mechanisms by which LGI1 controls synaptic activity. Fourthly, LE patient LGI1 antibodies may disrupt specific LGI1 interactions with ADAM proteins or other accessory proteins. Characterization of these interactions will provide additional insights into synaptic function. Curiously, one patient's serum was positive for LGI1 immunoreactivity, but negative in the immunoprecipitation assay for VGKCs, suggesting VGKC-independent functions for LGI1 (Irani et al., 2010). Thus, the identification of a connection between LGI1 and LE will advance both our understanding of synaptic biology, and our approach towards diagnosis and treatment of epilepsy.

## THE ROLE OF LGI2 IN CNS DEVELOPMENT

LGI2 is associated with canine Benign Familial Juvenile Epilepsy. In the Italian water dog *Lagotto Romagnolo*, the disease is transient, generally disappearing by 10 weeks of age, and could serve as a model for human remitting epilepsies. Analysis of a Finnish pedigree of these dogs indicated a primarily recessive inheritance (Jokinen et al., 2007). Using an expanded pedigree of these dogs, Seppälä and colleagues identified 11 pairs of discordant siblings (one affected, one not). Their DNA was subjected to genome-wide association analysis, revealing a region of homozygosity on canine chromosome 3. Subsequent sequencing revealed that roughly 36% of *Lagatto Romagnolo* dogs carry a point mutation in *LGI2* that results in truncation of 11 amino acids from the C-terminus of the mutant LGI2 protein (Seppälä et al., 2011), indicating that *LGI2*, like *LGI1*, is an epilepsy gene. Structurally, this mutation is adjacent to the frameshift mutation in *LGI1* in ADPEAF patients described by Kalachikov and colleagues (Kalachikov et al., 2002), providing additional evidence for the requirement of the C-terminal amino acids of LGI proteins to hold the EPTP domain structure together, as predicted by the Velcro model of β-propeller folding.

The protein interactions of LGI2 are less well studied than are those of LGI1. In culture, LGI2 was found to bind to the cell surface of ADAM11-expressing cells, but not to cells that expressed either ADAM22 or ADAM23 (Özkaynak et al., 2010). However, a later study in rat brain showed that LGI2 was, like LGI1, co-immunoprecipitated with both ADAM22 and ADAM23 antibodies, suggesting that LGI2 interacts–at least indirectly–with these ADAM proteins (Seppälä et al., 2011). As mentioned above, a truncating mutation in *LGI2* causes benign juvenile epilepsy in dogs. Just like most mutations in *LGI1* causes the mutated protein to be retained in the cell and degraded, this mutation of LGI2 completely abolishes its secretion (Seppälä et al., 2011). These observations suggest that LGI1 and LGI2 function through a similar mechanism that affects synaptic maturation at different time points of postnatal nervous system development. Importantly, LGI2 expression in the brain is highest preceding axonal pruning and before the onset of epilepsy, suggesting that LGI2 acts during the network construction phase (Seppälä et al., 2011). LGI1 might then act during the pruning phase to ensure an electrically stable network to serve the rest of the animal's life, explaining why in *LGI2*-mutant dogs' epileptic episodes are only seen in young animals.

## MULTIPLE POTENTIAL FUNCTIONS OF LGI3

Unlike *Lgi1*, *Lgi2* and *Lgi4*, mutations in *Lgi3* have yet to be associated with a pathological phenotype in humans or experimental animals. Mice in which *Lgi3* exon1 (including the initiation codon) has been deleted appear normal (Kim et al., 2013), although a residual 75 kD isoform suggests the existence of an alternative start site (Park et al., 2008; Kim et al., 2013). *In vitro* experiments using cell lines suggests that LGI3 may perform several distinct functions. Putative LGI3-specific antibodies co-immunoprecipitate syntaxin1, but not SNAP-25 or other components of the secretory apparatus (Park et al., 2008). Curiously, the C-terminus of syntaxin1 is buried within the plasma membrane, and is inaccessible (Suga et al., 2003). Therefore its interaction with LGI3 (a) occurs in the cytoplasm, (b) is indirect, perhaps mediated by ADAM proteins or (c) occurs as a result of tissue homogenization. The first two possibilities are reminiscent of the interaction between LGI1 and Kvβ1 (Schulte et al., 2006). Amyloid Aβ40 and Aβ42 peptides transiently up-regulate LGI3 expression in astrocytes, and LGI3 appears to promote Aβ endocytosis through an interaction with flotillin1 (Kimura et al., 2007; Okabayashi and Kimura, 2007, 2008, 2010). Thus LGI3 may mediate endocytosis for both syntaxin1 and amyloid peptides.

LGI3 may also function in cell types other than astrocytes. LGI3 expression in brain increases postnatally and it is expressed in neurons but not in oligodendrocytes (Lee et al., 2006; Okabayashi and Kimura, 2007), but it is enriched in homogenates of CNS myelin relative to homogenates of whole brain (Dhaunchak et al., 2010). These observations suggest that it may function in myelinated nerve fibres. Additionally, LGI3 induces neurite outgrowth and increases phosphorylation of the signal transduction proteins Akt and FAK (focal adhesion kinase) (Park et al., 2010). Keratinocytes express LGI3, and it may promote their survival following UV irradiation (Lee et al., 2012). Multiple neural crest-derived cell types express *Lgi3*, including melanoma cell lines (Rossi et al., 2005), DRG neurons (Bermingham et al., 2006) and adipocytes (Kim et al., 2012). LGI3 suppresses expression of the adipocyte hormone adiponectin (Kim et al., 2013), and it attenuates pre-adipocyte differentiation through binding to Adam23 (Kim et al., 2012). It is at present unclear what common LGI3-mediated mechanism might underlie these diverse biological functions. Together these observations suggest multiple functions for LGI3 that await further elucidation by the analysis of *Lgi3* mutant mice.

## INVOLVEMENT OF LGI4 IN CELL–CELL INTERACTIONS IN THE PERIPHERAL NERVOUS SYSTEM

Cell–cell interactions are required during PNS development for Schwann cell proliferation, migration, survival and myelination; however, the molecular mechanisms mediating these interactions are poorly understood. Study of the *claw paw* mutation in mice has revealed a novel LGI4-mediated signalling pathway that controls peripheral myelination. In 1977, Nelda Blaisdell, an animal technician handling C57BL/6-*obese* mice at The Jackson Laboratory in Bar Harbor, Maine, noted a litter in which two pups had limb abnormalities. Rather than holding their forelimbs up towards the head as most mouse pups do, they were held out, away from the body, or lowered towards the thorax. Mating of the littermates of these affected pups demonstrated that the phenotype was heritable. The new spontaneous mutation, called *claw paw* (*clp*) caused PNS hypomyelination without affecting central myelin (Henry et al., 1991). Because of the similarity in its myelination phenotype with that of mice lacking the POU domain transcription factor POU3f1/Oct6 (Bermingham et al., 1996; Jaegle et al., 1996), the *clp* mutation was investigated further. However, the *clp* mutation mapped to a separate locus on chromosome 7, and initial Oct6 expression was not affected (Darbas et al., 2004; Bermingham et al., 2006). The *clp* mutation was positionally cloned using a series of backcrosses, and found to result from an insertion in the *Lgi4* gene (Bermingham et al., 2006), demonstrating a critical role for LGI4 in peripheral myelination.

*Claw paw* (*clp/clp*) mice, in which LGI4 is not secreted (Bermingham et al., 2006), display a delay in radial sorting of axon fibres, and nerve-grafting experiments demonstrate that LGI4 function is required in Schwann cells and possibly in neurons (Darbas et al., 2004). LGI4 binds to ADAM22 (Table 1), and *Adam22* knockout mice show a similar peripheral hypomyelination and forelimb phenotype to *claw paw* mice (Sagane et al., 2005; Nishino et al., 2010; Özkaynak et al., 2010); peripheral hypomyelination also is seen following Schwann cell-specific deletion of *Lgi4*, and neuron-specific deletion of *Adam22* (Özkaynak et al., 2010). Thus, Schwann cell-secreted LGI4 appears to interact with ADAM22 on peripheral axons and in this way enable Schwann cell–neuron communication. The interaction could trigger a reciprocal ensheathment and/or myelination signal to Schwann cells (Figure 3). The nature of these putative signalling components is unknown. One possibility is that LGI4–ADAM22 modulates Nrg1 (neuregulin) signalling, but if so, it does not modulate the surface expression of Nrg1, a major regulator of Schwann cell migration and myelination (Özkaynak et al., 2010). Another possibility is that LGI4 modulates an already existing interaction between ADAM22 and another ADAM22-binding protein on the Schwann cell membrane, for example integrins (Figure 3). In addition to ADAM22, LGI4 can also bind to ADAM23 and ADAM11 (Sagane et al., 2008; Özkaynak et al., 2010). Whether these ADAM proteins play a role in PNS myelination is at present unknown. It is possible that–in analogy with the proposed role of LGI1 in regulating trans-synaptic adhesion (Figure 3)–LGI4 links axons and Schwann cells through interactions with ADAM22 in the axonal membrane and ADAM23 or ADAM11 in the Schwann cell membrane.

*Lgi4* is expressed initially by neural crest stem cells but its expression becomes restricted to the glial cells that derive from them. These neural crest-derived cells include, in addition to Schwann cells, enteric glial cells with properties resembling those of astrocytes in CNS, and satellite cells of (para-) sympathetic and dorsal root ganglia (reviewed in Jessen, 2004). Analysis of cultures derived from embryonic enteric and (para-) sympathetic ganglia revealed that LGI4 is required for proper levels of proliferation of glial precursors, but not for glial fate determination of neural crest stem cells (Nishino et al., 2010). Interestingly, embryonic sciatic nerve Schwann cells proliferated normally in the absence of LGI4 (Nishino et al., 2010), demonstrating that different types of PNS glial cells have different requirements for LGI4. Whether the proliferative effect of LGI4 on these glial precursors is mediated through an ADAM receptor is an important unanswered question. Thus, LGI4 has multiple functions including proliferation of enteric glia and satellite cells in PNS, and later, myelin formation in Schwann cells. The requirement for LGI4 for embryonic enteric glial cell and satellite cell proliferation contrasts with that of LGI1 for glioma cells as LGI1 inhibits proliferation of glioma cells (Chernova et al., 1998; Krex et al., 2002).

## CONCLUDING REMARKS

Over the last decade, LGI proteins have emerged as important regulators of cellular interactions in the nervous system and their mutation has been associated with diverse pathologies such as epilepsy, psychiatric disorders and hypomyelination. However, their mechanism or mechanisms of action remain a mystery. Advances in understanding one LGI family member may inform us about the functions of others. LGI proteins are secreted proteins, but because most mutations in LGI proteins affect their secretion, these mutations do not tell us much about the mechanism of action of LGI proteins. The disease-associated mutation in LGI1 that appears to affect its interactions with other proteins rather than its secretion (Striano et al., 2011) suggests that identification of additional disease-associated, yet normally secreted, LGI mutant proteins will help map their functionally relevant interfaces. Further insight into LGI protein function may come from studies that map the epitopes within LGI1 recognized by sera of different LE patients. It is not known at present whether the sera from different LE patients recognize the same or different epitopes. Mapping of these epitopes might tell us how these antibodies interfere with normal LGI1 function and cause disease. The crystal structure of an LGI protein will inform us of its overall shape, and how its functionally significant interfaces are oriented. These studies will provide a fuller understanding of LGI protein interactions with other proteins.

All LGI proteins appear to interact with ADAM22/23/11 receptors albeit probably with very different affinities. The high degree of identity among LGI proteins and their affinity for these ADAM receptors suggest that they act through a similar mechanism. However, it is not known whether the LGI proteins are functionally equivalent or serve distinct functions in different parts of the nervous system at different developmental stages. The one published experiment that addresses this issue demonstrated that LGI3 could not replace LGI1 in CNS synaptic development (Fukata et al., 2010), possibly due to a much lower affinity of LGI3 for the ADAM22 receptor. An understanding of the functional relationship of LGI proteins will require the determination of the relative affinities of these proteins for the ADAM receptors. Another open question is how LGI–ADAM interactions influence ADAM–integrin interactions: Are they mutually exclusive or does LGI binding potentiate ADAM–integrin interactions? Answering these questions, in addition to identifying the full repertoire of LGI receptors in different parts of the nervous system will elucidate mechanistic aspects that are common to the LGI protein family as well as to those that are member specific.

LGI proteins evolved with vertebrates, but many of the intercellular interactions in which they function, such as synapses between neurons, clearly predate them. Are the biological functions of LGI proteins required in invertebrates, and if so, what proteins perform them? If LGI proteins perform vertebrate-specific functions, could their appearance have been a crucial step in the evolution of complex vertebrate nervous systems? As we learn more about what LGI proteins do, the answers to these questions will become apparent.

Importantly, we anticipate that ongoing and future clinical, genetic and biochemical work directed towards an understanding of the biology and pathology of LGI proteins will ultimately lead to novel approaches in combating the devastating neurological diseases associated with mutations in the *LGI* gene family.
